# Effect of breathing exercise on stress management

**DOI:** 10.6026/973206300220425

**Published:** 2026-01-31

**Authors:** Resheetha Jeslin A, Thephilah Cathrine R, Shankar Shanmugam R, Rajathi S, Jayabharathi B

**Affiliations:** 1 Department of Pediatric Nursing, Meenakshi Academy of Higher Education & Research (MAHER), Chennai, India; 2 Department of Psychiatric Nursing, Meenakshi College of Nursing, Chennai, India; 3 Department of Child Health Nursing, College of Nursing, Madras Medical College (MMC), Chennai, India; 4 Department of Pediatric Nursing, Hindu Mission College of Nursing, Chennai, India; 5 Department of Obstetrics & Gynecological Nursing, Meenakshi College of Nursing, India

**Keywords:** Effectiveness, stress, breathing exercise, adolescents

## Abstract

Adolescence is a developmental phase marked by significant stress, which can negatively impact mental and physical well-being. Therefore, it is of interest to evaluate the effectiveness of breathing exercises as a stress management strategy for adolescents. The research involved 40 adolescents (aged 14-15 years) from schools in Tiruchirappalli, using the Modified Stress Scale for pretest and posttest data. Results showed a significant reduction in stress levels, with a paired t-test value of 8.1294 and a p-value of 0.0001. Thus, breathing exercises are an effective and accessible strategy for reducing stress in adolescents, addressing a gap in adolescent stress management methods.

## Background:

Adolescence is a crucial developmental phase marked by significant physical, emotional and social changes. These transformations, while part of the natural growth process, often bring about heightened levels of stress [1]. Adolescents face a variety of stressors that can significantly impact their mental, emotional and physical well-being. During this phase, young people encounter challenges such as academic pressure, body image concerns, family expectations and the complexities of social relationships. These stressors can affect their ability to perform academically, develop healthy social connections and maintain emotional balance [2]. Academic pressure is one of the most prominent stressors during adolescence. As students transition through middle and high school, they face an increasing workload, exams and the pressure to succeed [3]. This pressure can lead to feelings of inadequacy, anxiety and fear of failure, particularly as academic performance often becomes a measure of self-worth. Alongside academic demands, adolescents are navigating complex social dynamics [4]. Peer pressure, friendships, romantic relationships and the influence of social media can all be sources of stress. Social media, in particular, plays a powerful role in shaping adolescents' self-image and can lead to anxiety, depression and feelings of isolation when comparisons to others arise [5]. In addition to social and academic pressures, adolescents undergo physical changes due to puberty, which can lead to body image concerns. As their bodies change, many adolescents may experience discomfort with their appearance, leading to self-consciousness and a lack of confidence [6]. These changes, combined with emotional turbulence, make the adolescent years particularly vulnerable to stress. Family dynamics also play a significant role, as adolescents navigate their relationship with parents, siblings and other family members. Expectations related to family responsibilities, chores and the need for independence often creates tension in the home. Furthermore, challenges in family structure, such as divorce or separation, can exacerbate stress during this period [7]. Another contributing factor to adolescent stress is the process of self-discovery. Adolescents often struggle with finding their identity, balancing the desire for independence with the need for guidance. This search for personal identity can create confusion and frustration. Alongside these internal struggles, external stressors like bullying whether physical, verbal, or online add to the pressure adolescents experience [8]. Additionally, the societal expectations placed on them in terms of gender roles, career choices and future plans can further amplify stress. Trauma, abuse, neglect and the loss of loved ones are other significant stressors that affect adolescent well-being. Mental health issues such as anxiety and depression also contribute to the stress load, as adolescents may feel overwhelmed and unsure of how to seek help [9]. The impact of stress on adolescents can manifest in emotional symptoms such as irritability, anxiety, mood swings and depression. Physical symptoms such as headaches, stomachaches, fatigue and sleep disturbances are also common. Behavioral signs of stress may include substance abuse, withdrawal from social activities and engagement in risky behaviors. Left unaddressed, chronic stress can have long-term consequences on an adolescent's mental and physical health, potentially leading to issues like depression, anxiety, or substance use disorders. Educators, parents and mental health professionals must recognize the signs of stress and offer appropriate support and intervention to help adolescents build resilience and adopt effective coping mechanisms [10]. One effective strategy for managing stress among adolescents is the use of breathing exercises. Breathing exercises can help to reduce stress by calming the nervous system and lowering stress hormones like cortisol. These exercises trigger the body's relaxation response, leading to a sense of calm and improved emotional regulation. In addition, breathing exercises help adolescents stay focused and improve their attention, which can enhance performance in academic and social situations [11]. They also support emotional intelligence by providing adolescents with tools to understand better and regulate their emotions. Regular practice of breathing exercises can help build resilience, allowing adolescents to manage stress better and handle difficult situations. Furthermore, by promoting better sleep, these exercises contribute to overall well-being. Given their accessibility and ease of implementation, breathing exercises can be an invaluable tool for adolescents in managing daily stress and improving their overall mental health [12]. Therefore, it is of interest to determine the effectiveness of breathing exercises in helping adolescents manage stress, foster emotional resilience and improve their overall mental health and well-being.

## Methodology:

The objectives of this study are to assess the level of stress among adolescents, determine the effectiveness of breathing exercises in managing stress and examine the relationship between stress management levels and selected demographic variables. The study's hypotheses are as follows: NH1 posits that there is no significant difference in the level of stress among adolescents at the p < 0.05 level and NH2 suggests that there is no significant association between the level of stress and the selected demographic variables among adolescents at the p < 0.05 level. The research approach used in this study was a quasi-experimental pre-test design. The study was conducted in selected schools in the Tiruchirappalli district. A total of 40 adolescents, aged between 14 and 15 years, were included in the sample, selected using the convenience sampling technique. The inclusion criteria for sample selection were students in the 14-15 years age group, those who understood Tamil or English and those available at the time of data collection. The exclusion criteria included students who were unwilling to participate, those who were sick during data collection and those who had already undergone the program. The tool for data collection consisted of two parts. Part 1 collected demographic variables, including age, gender, father's education, mother's education, father's occupation, mother's occupation and socio-economic status, type of family, medium of studying and residential area. Part 2 utilized the Modified Adolescents' Assessment Stress Scale, which includes 24 items presented as statements with five alternatives on a Likert scale, scored from 5 to 1 respectively Table 1. Table 2 revealed that with regard to the age in years, the majority of the adolescents 29 (72.5%) were between the age group of 14 - 15 years and 11 (27.5%) were 13-14 years of age, with regard to birth order majority of them 24 (60%) were boys, 16 (40%) of them girls, with regard fathers education 19 (47.5%) of them completed Higher Secondary, 11 (27.5%) of them Secondary school, 7 (17.5%) of them degree, 3 (7.5%) no formal education, regarding mothers education 16 (40%) Secondary school, 14 (35%) Higher Secondary School, 8 (20%) degree and above, 2 (5%) no formal education, with regard fathers occupation majority of them 21 (52.5%) self-employment, 15 (37.5%) holding private employment, 4 (10%) government employees, with regard mothers occupation majority of them 28 (70%) house wife, 6 (15%) self-employment, 4 (10%) private employment, 2 (5%) government employee, with regard to socio economic status majority of them 18 (45%) middle class, 10 (25%) lower class, 9 (22.5%) upper middle class, 3 (7.5) upper class, with regard medium of school majority 24 (60%) English medium, 16 (40%) Tamil medium, with regard birth order majority of them 27 (42.5%) second birth order, 16 (40%) first birth order, 7 (17.5%) third birth order and with regard area of residency majority of them 26 (65%) belongs to rural, 8 (20%) urban and 6 (15%) semi urban. Table 3 presents the results of the analysis of the effectiveness of deep breathing exercises on stress management among adolescents. The mean stress score decreased from 65.325 (SD = 19.861) in the pretest to 61.125 (SD = 18.436) in the posttest. The paired t-test yielded a value of 8.1294 with 39 degrees of freedom and a highly significant p-value of 0.0001 (p < 0.05), indicating a statistically significant reduction in stress levels after the intervention. These results demonstrate that deep breathing exercises effectively reduce stress among adolescents, supporting their use as a beneficial and accessible stress management technique.

Table 4 presents the Chi-Square tests conducted on various demographic variables revealed no significant associations with stress levels among the participants. For the variable of age, the Chi-Square statistic was 1.96 with a p-value of 0.375, indicating no significant association. Similarly, the analysis of gender yielded a Chi-Square statistic of 5.18 and a p-value of 0.075, again showing no significant relationship. The socioeconomic status variable produced a Chi-Square statistic of 2.09 with a p-value of 0.911, confirming the absence of significant association. Additionally, the medium of school demonstrated a Chi-Square statistic of 0.72 and a p-value of 0.699, indicating no significant correlation. The birth order variable also showed no significant association with a Chi-Square statistic of 3.98 and a p-value of 0.408. Lastly, the area of residence had a Chi-Square statistic of 1.23 with a p-value of 0.874, reinforcing the finding of no significant association.

## Discussion:

The results of this study, showing a significant reduction in adolescent stress levels post-breathing exercises, align closely with findings from several previous studies exploring similar interventions. Bentley *et al.* (2023) [11] demonstrated that various stress-reduction techniques, including deep breathing, significantly decreased anxiety and improved emotional regulation in adolescents. This parallels the findings of this study, where breathing exercises showed a significant decrease in stress scores. Similarly, Collado-Soler *et al.* (2023) [12] focused on adolescent stress management through mindfulness and relaxation techniques, concluding that such interventions could lead to improved emotional well-being and stress reduction . This aligns with the current study's findings, as deep breathing exercises, a form of relaxation technique, were effective in managing stress. Mahalakshmi *et al.* (2024) [13] explored the effects of structured relaxation and mindfulness exercises on adolescents, noting a reduction in perceived stress levels. This supports the idea that structured breathing exercises, which are simple yet effective, are valuable tools in stress management strategies for adolescents. The results from the current study contribute to this broader understanding by showing that even simple techniques like deep breathing can significantly reduce stress without the need for complex interventions. Balban *et al.* (2023) [14] highlighted the importance of mindfulness and breath-focused exercises in reducing chronic stress in vulnerable adolescent populations. This finding underscores the effectiveness of deep breathing in promoting long-term resilience, which is a key outcome in the current study. Although demographic factors such as age, gender and socio-economic status did not show a significant correlation with stress reduction in this study, similar studies have emphasized that mindfulness interventions like deep breathing have broad applicability across different demographic groups.

## Conclusion:

Deep breathing exercises are an effective and accessible method for reducing stress among adolescents. The significant reduction in stress levels post-intervention underscores its potential as a valuable stress management tool. Despite no significant associations with demographic variables, the simplicity and effectiveness of deep breathing make it a practical technique for enhancing adolescent well- being and resilience.

## Figures and Tables

**Table 1:** Interpretation of the score

**Sl.no**	**Interpretation**	**Score**
1	Mild level of stress	<40
2	Moderate level of stress	4 1 - 80
3	Severe level of stress	81 - 120

**Table 2:** Frequency and percentage distribution of demographic variables

**Sl. No.**	**Demographic variables**	**Frequency (n)**	**Percentage (%)**
**1**	**Age**		
	a)13-14	11	27.50%
	b)14-15	29	72.50%
**2**	**Gender**		
	a)Boys	24	60%
	b)Girls	16	40%
**3**	**Father's education**		
	a)No formal education	3	7.50%
	b)Secondary school	11	27.50%
	c)Higher secondary school	19	47.50%
	d)Degree and above	7	17.50%
**4**	**Mothers' education**		
	a)No formal education	2	5%
	b)Secondary school	16	40%
	c)Higher secondary school	14	35%
	d)Degree and above	8	20%
**5**	**Father's occupation**		
	a)Self-employment	21	52.50%
	b)Private	15	37.50%
	c)Government	4	10%
**6**	**Mother's occupation**		
	a)Self-employment	6	15%
	b)Private	4	10%
	c)Government	2	5%
	d)House wife	28	70%
**7**	**Socio economic status**		
	a)Upper class	3	7.50%
	b)Upper middle class	9	22.50%
	c)Middle class	18	45%
	d)Lower class	10	25%
**8**	**Medium of school**		
	a)English	24	60%
	b)Tamil	16	40%
**9**	**Birth order**		
	a)First	16	40%
	b)Second	27	42.50%
	c)Third	7	17.50%
**10**	**Area of residence**		
	a)Rural	26	65%
	b)Urban	8	20%
	c)Semi-urban	6	15%

**Table 3:** Effectiveness of Deep Breathing exercise on stress management among adolescents

**Stress score**	**Mean**	**SD**	**Paired 't' test**	**df**	**'p' value**
Pretest	65.325	19.861	8.1294	39	0.0001**
Posttest	61.125	18.436			
**significant at
(p<0.0001) level

**Table 4:** Association with selected demographic variables

**Variable**	**Chi-Square**	**p-value**	**Significance**
Age	1.96	0.375	No significant association
Gender	5.18	0.075	No significant association
Socioeconomic Status	2.09	0.911	No significant association
Medium of School	0.72	0.699	No significant association
Birth Order	3.98	0.408	No significant association
Area of Residence	1.23	0.874	No significant association
